# RETRACTION: Potential Therapeutic Role of Z-Isochaihulactone in Lung Cancer through Induction of Apoptosis via Notch Signaling

**DOI:** 10.1155/ecam/9894619

**Published:** 2025-06-12

**Authors:** Evidence-Based Complementary and Alternative Medicine

RETRACTION: J.-P. Ou, H.-Y. Lin, K.-Y. Su, et al., “Potential Therapeutic Role of Z-Isochaihulactone in Lung Cancer through Induction of Apoptosis via Notch Signaling,” *Evidence-Based Complementary and Alternative Medicine* 2012, no. 1 (2012): 1–11, https://doi.org/10.1155/2012/809204.

The above article, published online on 24 September 2012 in Wiley Online Library (https://wileyonlinelibrary.com), has been retracted by John Wiley & Sons Ltd.

The retraction has been agreed following an investigation of concerns raised by *Actinopolyspora biskrensis* on PubPeer [1]. The investigation has identified similarities between the caspase-3 bands in Figure 2(c) and the cleaved caspase-3 bands in Figure 2(d). The investigation has also identified concerns related to Figure 4(d), where both sets of bands appear similar to the bands shown in Figure 1(b) of [2], in which they are attributed to different biomarkers.

As a result of the investigation, the data and conclusions of this article are considered unreliable.

The authors have been informed of the retraction but did not provide a response.
